# *Bacillus amyloliquefaciens* SC06 Ameliorated Intestinal Mucosal Injury by Regulated Intestinal Stem Cells Proliferation and Differentiation via Activating Wnt/β-Catenin Signal Pathway in *Clostridium perfringens*-Challenged Mouse

**DOI:** 10.3390/microorganisms13092136

**Published:** 2025-09-12

**Authors:** Hongbin Deng, Si Cheng, Jiemei Fan, Haibin Hao, Dandong Fang, Weiqin Li, Qi Wang

**Affiliations:** 1 Department of Critical Care Medicine, Jinling Hospital, Affiliated Hospital of Medical School, Nanjing University, Nanjing 210000, China; hongbindeng@yeah.net (H.D.); chengsi163@yeah.net (S.C.); aiguoshu3@163.com (J.F.); haoweipei104@163.com (H.H.); fangdandong96@163.com (D.F.); 2 Institute of Animal Nutrition and Feed Sciences, College of Animal Sciences, Zhejiang University, Hangzhou 310058, China

**Keywords:** *Bacillus amyloliquefaciens* SC06, Necrotic enteritis, *Clostridium perfringens*, jejunum organoids, Intestinal stem cells (ISCs), Wnt/β-catenin pathway

## Abstract

The objective of our study was to verify the intervention effect of *Bacillus amyloliquefaciens* SC06 on NE by constructing a *C. perfringens*-induced intestinal damage mouse model. A total of 40 mice were randomly assigned to four treatments: CON (basal diet), CP (basal diet + *C. perfringens*), SC06 + CP (basal diet + SC06 + *C. perfringens*) and SC06 (basal diet + SC06). Our findings indicated that SC06 supplementation was effective in maintaining the integrity of the intestinal barrier, enhancing the antioxidant capacity of the intestine, reducing the generation of an inflammatory response, and suppressing enterocyte apoptosis in the presence of *C. perfringens*. Furthermore, SC06 supplementation enhanced the prefoliation of intestinal stem cells (ISC) and prompted their differentiation into goblet cells and Paneth cells. Moreover, our findings indicate that SC06 promotes the proliferation of *C. perfringens*-induced jejunum organoids and the expression of genes and proteins associated with ISC differentiation and regeneration. The mechanism by which SC06 modulates ISCs has been validated, and the results align with those obtained in vivo. In conclusion, the findings demonstrated that SC06 stimulates the proliferation and differentiation of ISCs through the activation of the Wnt/β-catenin signaling pathway, thereby accelerating epithelial regeneration and repair.

## 1. Introduction

*Clostridium perfringens* (CP) is a widespread environmental bacterium and a component of the normal intestinal flora of humans and animals [1,2,3,4]. Necrotic enteritis (NE) caused by *C. perfringens* has a global reach, with cases reported in the United Kingdom, the United States and China [5,6,7]. The disease is prevalent in these countries due to the high incidence of infection and cross-contamination through *C. perfringens* [8,9]. The objective of clinical treatment for NE is to achieve mucosal restoration, which is the gold standard for achieving long-term remission [10]. The process of regenerating and restoring the intestinal epithelium is an integral part of the mucosal repair procedure [11]. It is therefore imperative that damage to the intestinal epithelium be repaired to prevent injury to the intestinal mucosa in NE. Several studies have demonstrated that intestinal stem cells are indispensable for the functional recuperation and restoration of the compromised intestinal mucosal barrier. The self-renewal and proliferation of these cells are vital for the healing of injuries sustained by the digestive tract [12]. Following an external injury to the intestine, the repair function of stem cells is initiated by appropriate molecular mechanisms that facilitate the proliferation of ISCs and the production of progeny cells to replace damaged intestinal epithelial cells and regenerate the intestinal barrier [13,14]. The regeneration and restoration of the intestinal epithelium are contingent upon the equilibrium between intestinal stem cell (ISC) differentiation and renewal [15].

*Bacillus amyloliquefaciens* (*B. amyloliquefaciens*) is a potentially useful and multifunctional bacterium that can be employed in the synthesis of food ingredients, hydrolysis and fermentation [16,17]. Chen et al. [18] reported that *B. amyloliquefaciens* has the capacity to alter microbial composition and enhance host-microbe interactions, thereby directly improving the intestinal barrier in DSS-challenged mice. Our previous research has demonstrated that *B. amyloliquefaciens* SC06 exerts anti-inflammatory effects in obese mice through mechanisms such as enhancing intestinal barrier function and modulating the gut microbiota [19]. Nevertheless, the available evidence suggests that *B. amyloliquefaciens* SC06 may facilitate epithelial regeneration and repair from an ISC perspective in NE, although further investigation is required to substantiate this claim.

In the present study, the therapeutic effect of *B. amyloliquefaciens* SC06 on NE was assessed using a *C. perfringens*-induced NE mouse model. The objective was to investigate how *B. amyloliquefaciens* SC06 might facilitate mucosal healing by accelerating epithelial repair and regeneration via ISC differentiation and renewal. Furthermore, we constructed a jejunum organoid inflammatory injury model induced by *C. perfringens* to assess the capacity of *B. amyloliquefaciens* SC06 to repair epithelial damage and confirm the underlying mechanisms.

## 2. Materials and Methods

### 2.1. Animal and Probiotic Strain

The *B. amyloliquefaciens* SC06 strain, which has been assigned the number (CCTCC No: M2012280) by the China Center for Type Culture Collection, was isolated from soil. *C. perfringens* type A (ATCC 13124) was cultured in Reinforced clostridium medium (RCM; Hopebio, Qingdao, China) and incubated at 37 °C under anaerobic conditions for 18 h. The bacterial cultures that had been incubated overnight were centrifuged at 5000× *g* for 5 min. Using a standard curve, the concentrations of bacteria were determined after they were resuspended in sterile phosphate-buffered saline (PBS, pH = 7.2) and rinsed three times. Then, they were diluted to a specific concentration and stored at 4 °C for future use. A total of 40 healthy male C57BL/6 mice (18–22 g, 4 weeks old) were purchased from the Experimental Animal Center of Zhejiang Province (Hangzhou, China). C57BL/6 mice were maintained in enclosures at a temperature of 23 ± 2 °C and a relative humidity of 55 ± 5%, with a 12 h light/dark cycle.

**Figure 1:**
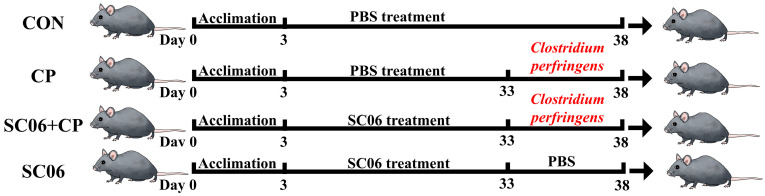
Experimental design and animal groups.

After acclimatization for 3 days, mice were randomly divided into 4 groups (n = 10): the control (CON, orally administered physiological saline), *C. perfringens* group (CP, administered orally with physiological saline 30 days, and 1 × 10^8^ CFU *C. perfringens* 5 days), *B. amyloliquefaciens* SC06 group (SC06, orally administered 1 × 10^8^ CFU *B. amyloliquefaciens* 35 days), *B. amyloliquefaciens* SC06 + *C. perfringens* group (SC06 + CP, orally administered 1 × 10^8^ CFU *B. amyloliquefaciens* 30 days, and 1 × 10^8^ CFU *Clostridium perfringen* 5 days). The experimental design is illustrated in the accompanying schematic in Figure 1. On the 38th day of the experiment, all mice were fully anesthetized in an ether container. Serum was collected from the heart and subsequently subjected to centrifugation at 3000× *g* for 15 min at 4 °C. The samples were subsequently stored at −80 °C until analysis. The body weights of the mice were monitored daily throughout the course of the experiment to assess any changes that might have occurred. The animal experiments in this study were allowed by Laboratory Animal Welfare and Ethics Committee of Zhejiang University (Ethical Code License No. ZJU20230360).

### 2.2. Biochemical Assays

The concentrations of serum TNF-α (tumor necrosis factor), IL-1β (interleukin-1β), IL-6 (interleukin-6), and IL-8 (interleukin-8), D-LA (D-lactic acid), DAO (diamine oxidase), and endotoxin were quantified using assay kits (Nanjing Jiancheng Bioengineering Institute, Nanjing, China).on a spectrophotometer (SpectraMax M5, Molecular Devices, Sunnyvale, CA, USA) in accordance with the manufacturer’s instructions.

### 2.3. Hematoxylin and Eosin (H&E) Staining

An analysis of the morphological characteristics of the intestinal tissue was conducted by dehydrating the specimens using a series of ethanol and xylene solutions. This was performed after the tissue had been fixed in a paraformaldehyde solution for a period of 24 h at room temperature. Subsequently, the samples were processed into paraffin blocks. Each specimen was cut into 5 μm cross-sections and stained with haematoxylin and eosin (Sigma-Aldrich, St. Louis, MO, USA). Subsequently, the segments were dehydrated for 10 min with 90% and 100% ethanol solutions, respectively. Subsequently, the tissues were examined, and images were captured using a microscope (BX63, Olympus, Tokyo, Japan) following the application of cedar oil for sealing. Concurrently, measurements were taken of the villus height and crypt depth.

### 2.4. Morphology of Jejunum Microvilli

Experiments using transmission electron microscopy (TEM) were performed on jejunum tissues after they were first fixed in 1% hungry acid and then submerged in a glutaraldehyde solution that contained 2.5% glutaraldehyde. In a nutshell, the samples were dehydrated by means of a succession of graded alcohols, which were then replaced with acetone, and then those samples were embedded in Epon 812 resin (SPI Supplies Inc., West Chester, PA, USA). Uranyl acetate citrate was used in a twofold staining method so that the sections could be examined more closely. The sample sections were observed using a Hitachi H-7650 transmission electron microscope (TEM) (Hitachi, Ibaraki, Japan) and photographed with a Gatan 830 charge-coupled device (CCD) camera (Gatan, CA, USA).

### 2.5. Crypt Isolation and Intestinal Organoid Culture

The preparation of intestinal organoids was carried out in accordance with the methods that Mahé et al. Disclosed [20]. After the mouse jejunum had been dissected and fragmented, the supernatant was washed with DPBS twenty times to remove any remaining debris. The pieces were then put in a solution of DPBS that included 2.5 mM EDTA, and the mixture was incubated on ice for a period of 30 min. Using centrifugation at a mass of 290× *g* for a period of 5 min, the crypt fractions were separated. This was performed after the combination of intestinal pieces was vortexed and then subjected to filtration using a cell strainer with a size of 70 µm. Rudimentary crypts were generated at 200× *g* for 3 min to eradicate individual cells. Matrigel (BD Bioscience/Corning, New York, NY, USA) and IntestiCultTM rganoid Growth Medium (Stemcell Technologies, Vancouver, BC, Canada) were equal volumes that were used to plate the crypts on 24-well plates. In an incubator containing 5% CO_2_ and 37 °C, the culture plate was incubated for fifteen minutes. After Matrigel had solidified, IntestiCultTM Organoid Growth Medium was applied. Organoid studies were treated as follows: CON (basic medium), SC06 (1 × 10^8^ CFU/mL SC06 treatment 24 h), CP (1 × 10^8^ CFU/mL *C. perfringens* treatment 6 h), SC06 + CP (1 × 10^8^ CFU/mL SC06 pre-treatment 24 h, then 1 × 10^8^ CFU/mL *C. perfringens* treatment 6 h). The jejunum organoids’ measurements were ascertained from an earlier description [21]. After calibrating the scale bar, the organoids’ areas were measured manually using ImageJ software (V1.8.0, NIH, Bethesda, MD, USA).

### 2.6. Total RNA Extraction, Reverse Transcription, and Relative Quantitative Real-Time PCR

The TRIZOL reagent (TaKaRa, Tokyo, Japan) was effectively used to isolate total RNA from the jejunal mucosa and organoids. Agar gel electrophoresis was used to assess the RNA’s integrity. In accordance with the instructions provided in the manufacturer’s manual, the PrimeScript II 1st Strand cDNA Synthesis Kit (Vazyme, Nanjing, China) should be utilized to synthesize cDNA. In accordance with the instructions provided by the manufacturer, the total RNA was reverse-transcribed using a PrimeScript RT reagent kit (Vazyme, Nanjing, China). Subsequently, the cDNA was stored at −20 °C. Utilizing the HiScript II One Step RT-qPCR SYBR Green Kit (Vazyme, Nanjing, China) and the StepOne Plus Real-Time PCR system (Applied Biosystems, Carlsbad, CA, USA) to performer RT-qPCR analysis. To design the primers utilized in this investigation, the NCBI Primer-Blast tool was applied (Table 1). For the purpose of the control, a value that was equivalent to the mean of β-actin was used. The measurement of the relative abundance of expression for each target gene was carried out by using the 2^−ΔΔCt^ technique, as was previously reported in reference number [22].

### 2.7. Immunofluorescence and Tunel Analysis

Immediately after a fixation period of five minutes in cold methanol, the samples were blocked for two hours at a temperature of twenty-six degrees Celsius with 2.5% bovine serum albumin (BSA). The samples were then subjected to a 12 h incubation at a temperature of 4 °C with the appropriate antibody (Ki67, MUC2, LYZ, Abcam, Cambridge, MA, USA). A solution of 4′,6-diamidino-2-phenylindole (DAPI) and an antibody that was conjugated with Alexa Fluor 488 (Biotime Biotechnology, Xiamen, China) were used to stain the nuclei and the cells. Through the use of a confocal microscope (BX61W1-FV1000, manufactured by Olympus Corporation in Tokyo, Japan), images were acquired.

The preparation and staining of the jejunum section were accomplished with the use of a commercial TdT-mediated dUTP Nick-End labeling (TUNEL) kit manufactured (Fluorescein, Basel, Switzerland). When using fluorescence microscopy, it was possible to identify TUNEL-positive nuclei by their green fluorescence. On the other hand, all of the other nuclei in the jejunum were shown to have a blue fluorescence. At a high magnification of 200 times, the pictures were obtained, and for the purpose of statistical analysis, a total of five sections were chosen from each of the groups. In order to determine the apoptotic index, it was necessary to multiply the number of nuclei that were positive for TUNEL by the total number of nuclei [23].

### 2.8. Western Blot Analysis

Colon tissue lysates prepared with RIPA buffer underwent BCA protein quantification (Sigma, Saint Louis, MO, USA). Equal protein concentrations were separated via 12% SDS-PAGE and electrotransferred onto PVDF membranes (Millipore, MA, USA). Membranes were blocked with 5% skim milk (2 h, 26 °C) and subsequently incubated with primary antibodies overnight at 4 °C against: β-catenin, FZD7 and β-actin (Cell Signaling Technology, Danvers, MA, USA). After five TBST washes, HRP-conjugated secondary antibodies were applied (2 h, 26 °C). Protein bands were visualized using a Millipore chemiluminescent HRP substrate kit (Burlington, MA, USA) imaged on a Tanon system (China). Band intensities quantified by ImageJ 1.8.0 software normalized protein levels to β-actin.

### 2.9. Statistical Analysis

One-way ANOVA and multiple comparison tests were performed using SPSS software (version 20.0; IBM Inc., New York, NY, USA) to evaluate Turkey’s method of data detection. The mean values were reported with their respective standard errors (SD), and *p* < 0.05 was considered to indicate a statistically significant difference. The graphing software utilized was GraphPad Prism 8.4.2.

## 3. Results

### 3.1. B. amyloliquefaciens SC06 Improved the Growth Performance and Intestinal Integrity of C. perfringens-Challenged Mouse

**Figure 2:**
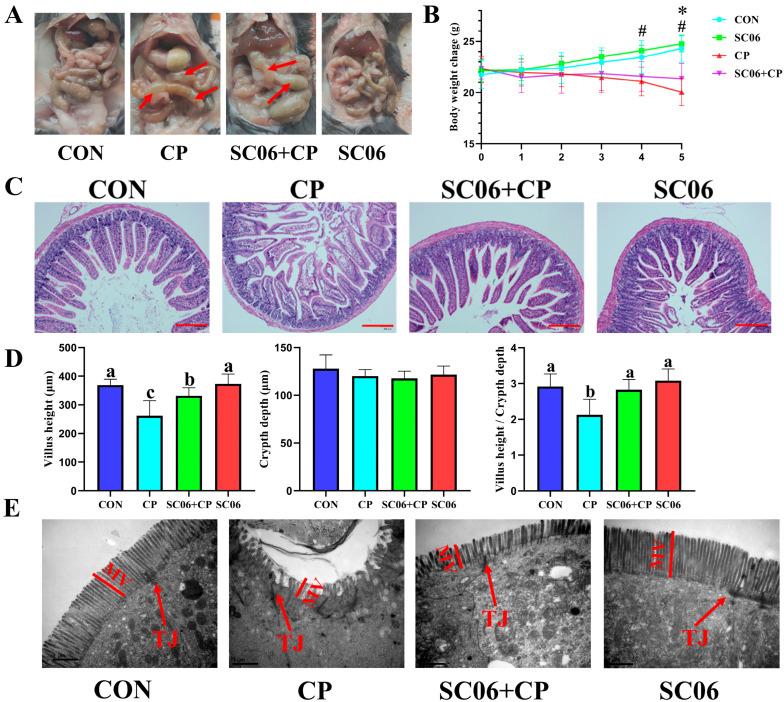
Effects of SC06 on the intestinal physical barrier function in *C. perfringens*-challenged mouse. (**A**) Intestinal damage status. (**B**) Body weight change. # *p* < 0.05; * *p* < 0.05. (**C**,**D**) Histomorphology of the jejunum. (**E**) TEM of the jejunum microvilli. TJ, tight junction. MV, microvilli length. Scale bars, 200 μm. Means ± SD (n = 6). ^a–c^ Means within a row with various superscripts vary substantially (*p* < 0.05).

**Figure 3:**
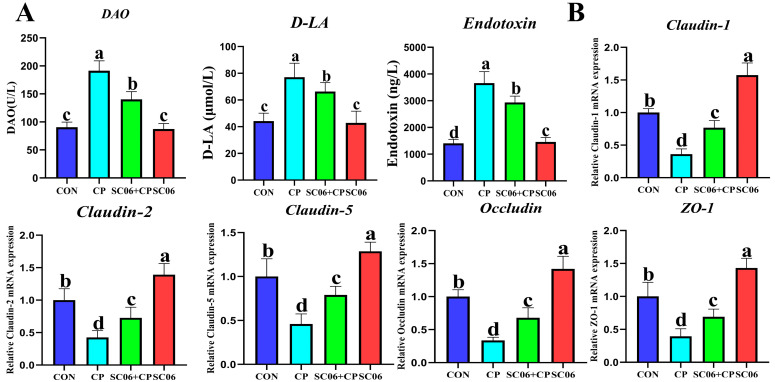
Effects of SC06 on the expression of jejunum tight junction proteins and serum intestinal barrier markers in *C. perfringens*-challenged mouse. (**A**) The content of DAO, D-LA and Endotoxin. (**B**) The gene expression of *Claudin-1*, *Claudin-2*, *Claudin-5*, *Occludin* and *ZO-1*. Means ± SD (n = 6). ^a–d^ Means within a row with various superscripts vary substantially (*p* < 0.05).

The NE mouse model was developed through the administration of *C. perfringens* treatment. On day 38, the mice that had been treated with *C. perfringens* exhibited significant (*p* < 0.05) intestinal bloating and weight loss (Figure 2A,B), confirming the successful modeling of the disease. Administration of SC06 reversed this trend. Histological examination by H&E staining demonstrated that SC06 significantly (*p* < 0.05) increase in villus height and V/H in mice subjected to *C. perfringens* challenge (Figure 2C,D). Additionally, transmission electron microscopy (TEM) revealed that the SC06 + CP group exhibited significantly (*p* < 0.05) longer microvilli than the CP group (Figure 2E). Additionally, we observed that *C. perfringens* markedly (*p* < 0.05) elevated the levels of D-LA and endotoxin, as well as the activity of DAO. However, SC06 effectively reversed this trend (Figure 3A). The qPCR results also indicated that *C. perfringens* significantly (*p* < 0.05) decreased the gene expression of *Claudin-1*, *Claudin-2*, *Claudin-5*, *Occludin*, and *ZO-1*. In contrast, SC06 treatment reversed this trend (Figure 3B).

### 3.2. B. amyloliquefaciens SC06 Suppressed the Levels of Inflammatory Cytokines and Oxidative Stress in C. perfringens-Challenged Mouse

**Figure 4:**
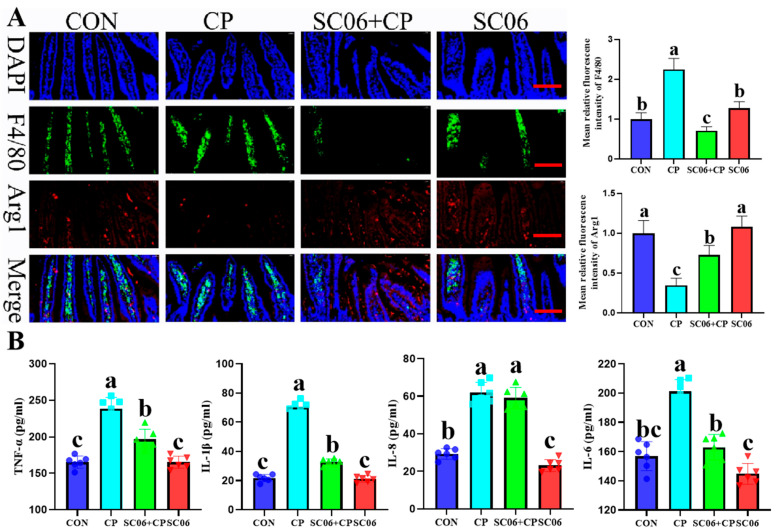
Effects of SC06 on the content of cytokines and antioxidant indicators in *C. perfringens*-challenged mouse. (**A**) F4/80 and Arg1 immunofluorescence analysis in the jejunum. (**B**) the content of TNF-α, IL-1β, IL-8 and IL-6. Scale bars, 100 μm. Means ± SD (n = 6). ^a–c^ Means within a row with various superscripts vary substantially (*p* < 0.05).

**Figure 5:**
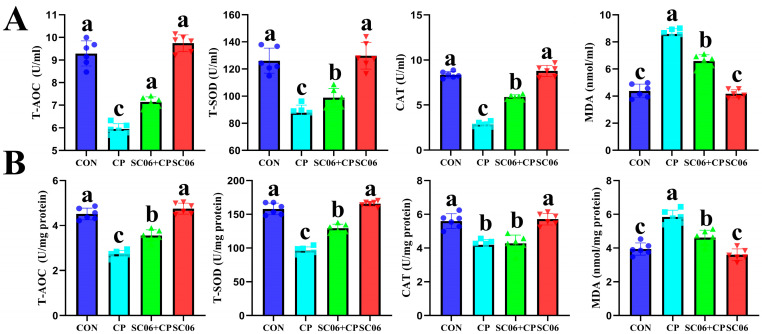
Effects of SC06 on the content of antioxidant indicators in *C. perfringens*-challenged mouse. (**A**,**B**) Serum and jejunum antioxidant indicators (T-AOC, T-SOD, CAT and MDA). Scale bars, 100 μm. Means ± SD (n = 6). ^a–c^ Means within a row with various superscripts vary substantially (*p* < 0.05).

As illustrated in Figure 4A, in comparison to the CON group, the mean fluorescence intensity of F4/80 (a marker for M1 macrophages) increased significantly (*p* < 0.05), and the mean fluorescence intensity of Arg1 (a marker for M2 macrophages) decreased significantly (*p* < 0.05) in response to the CP challenge. These changes were reversible with SC06 supplementation (*p* < 0.05). In addition, SC06 markedly (*p* < 0.05) diminished the concentrations of TNF-α, IL-1β, IL-6, and IL-8 in comparison to the CP group (Figure 4B). Due to the fact that there is a well-established connection between oxidative stress in the jejunum and the onset of inflammatory disorders, a deficit in mucosal antioxidant defense is a crucial component that contributes to the severity of NE. The level of MDA, and activities of CAT, GSH, and SOD in the jejunum and serum demonstrated that SC06 significantly (*p* < 0.05) attenuated the oxidative stress markers induced by *C. perfringens* (Figure 5).

### 3.3. B. amyloliquefaciens SC06 Decreased the Intestinal Cell Apoptosis Rates of Cells in C. perfringens-Challenged Mice

**Figure 6:**
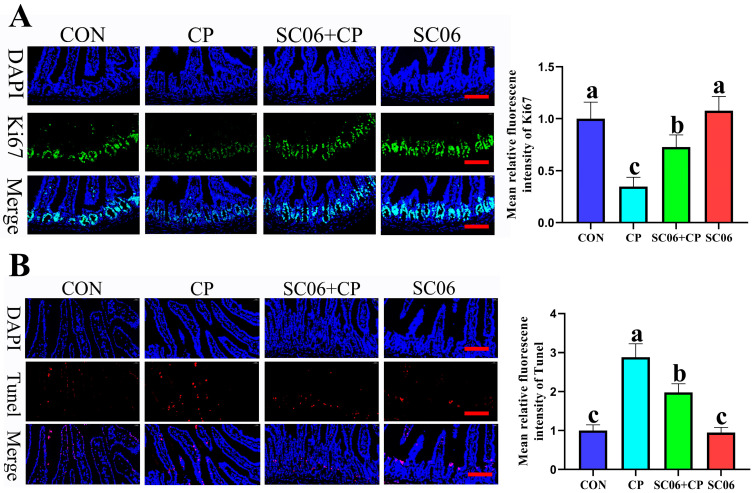
Effects of SC06 on jejunum apoptosis status in *C. perfringens*-challenged mouse. (**A**) Ki67 immunofluorescence analysis in the jejunum. (**B**) TUNEL immunofluorescence analysis in the jejunum. Scale bars, 100 μm. Means ± SD (n = 6). ^a–c^ Means within a row with various superscripts vary substantially (*p* < 0.05).

On the jejunum, the presence of *C. perfringens* resulted in a substantial increase (*p* < 0.05) in the apoptotic rate and an inhibition of cell growth. However, the addition of SC06 resulted in the restoration of these effects. As shown in Figure 6A,B, a comparison of the CON group with the SC06 group revealed that there were no significant changes (*p* > 0.05) in the expression of the Ki67 protein between the CON and SC06 groups.

### 3.4. B. amyloliquefaciens SC06 Promoted ISC Proliferation and Differentiation in C. perfringens-Challenged Mice

**Figure 7:**
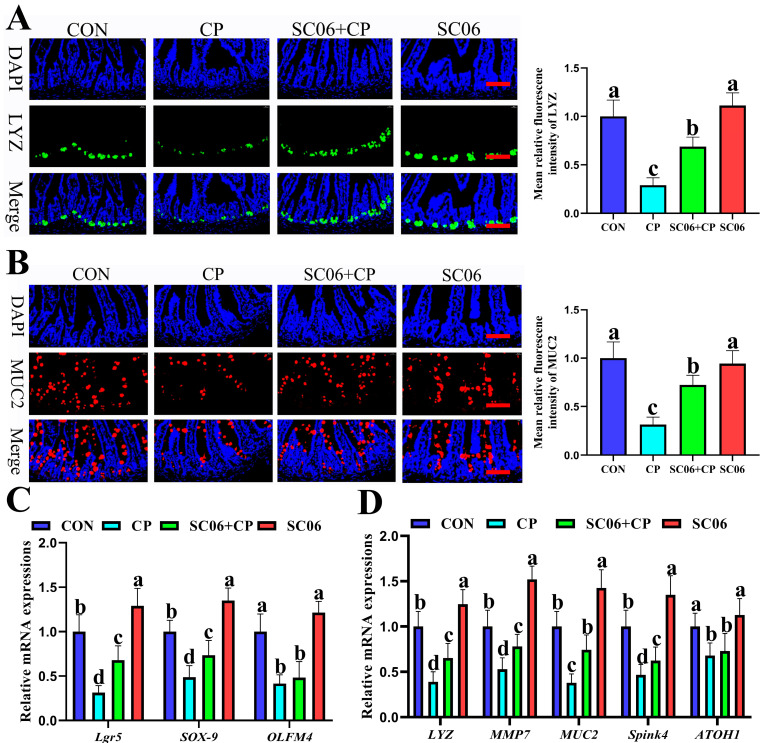
Effects of SC06 on jejunum stem cell proliferation and differentiation of *C. perfringens*-challenged mouse. (**A**,**B**) Immunofluorescence analysis of LYZ and MUC2. (**C**) the relative mRNA of Intestinal stem cell marker genes (*Lgr5*, *Sox-9* and *Olfm4*). (**D**) the relative mRNA of Paneth cell marker genes (*LYZ* and *MMP7*) and goblet cell marker genes (*MUC2*, *ATOH1* and *Spink4*). Scale bars, 100 μm. Means ± SD (n = 6). ^a–d^ Means within a row with various superscripts vary substantially (*p* < 0.05).

The results of the immunofluorescence analysis targeting the Paneth cell marker (LYZ) and goblet cell (MUC2) indicated that the treatment with SC06 significantly increased (*p* < 0.05) the number of Paneth cells and goblet cells in the jejunum when compared to the CP group (Figure 7A,B). Similarly, SC06 also significantly (*p* < 0.05) increased the mRNA expression levels of intestinal stem cell (ISC) markers (*Lgr5*, *Olfm4*, and *Sox9*), Paneth cell markers (*LYZ* and *MMP7*), and goblet cell markers (*MUC2*, *ATOH1*, and *Spink4*) when compared to the CP group (Figure 7C,D).

### 3.5. B. amyloliquefaciens SC06 Activated the Wnt/β-Catenin Signaling Pathway in C. perfringens-Challenged Mice

**Figure 8:**
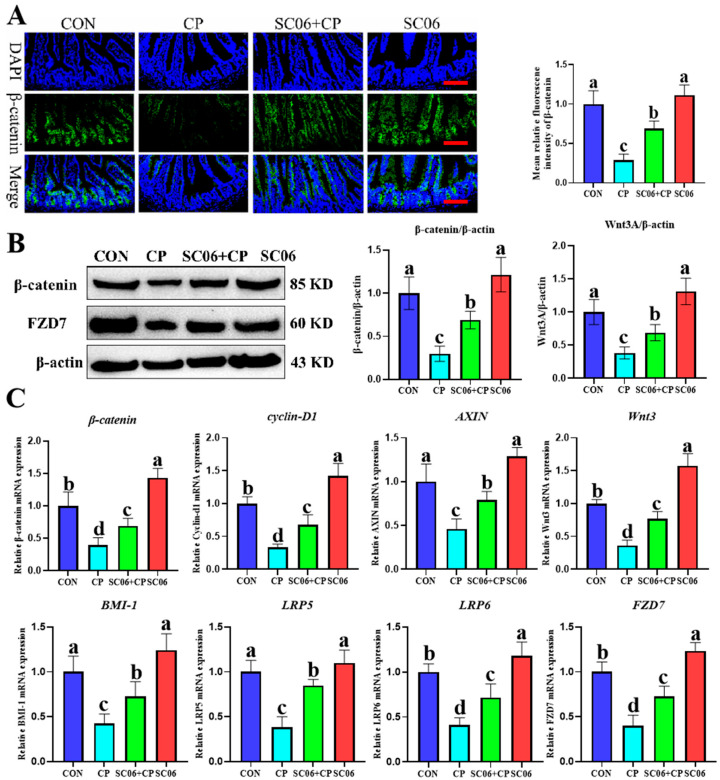
Effects of SC06 on the activation of the Wnt/β-catenin signaling pathway in *C. perfringens*-challenged mouse. (**A**) Immunofluorescence analysis of β-catenin in jejunum. Scale bars, 100 μm. (**B**) Western blot analysis of β-catenin, Cyclin-D1, and C-myc protein expression in jejunum. Means ± SD (n = 6). (**C**) Relative mRNA expression of Wnt/β-catenin signaling-related genes, including β-catenin, Cyclin-D1, AXIN, Wnt3, BMI-1, LRP5, LRP6, and FZD7, as determined by qPCR. ^a–d^ Means within a row with various superscripts vary substantially (*p* < 0.05).

The results of the immunofluorescence analysis demonstrated that *C. perfringens* significantly (*p* < 0.05) decreased the protein expression of β-catenin. In contrast, treatment with SC06 reversed this trend (Figure 8A). Furthermore, Western blot analysis revealed that C. perfringens significantly (*p* < 0.05) reduced the protein expression of β-catenin, Cyclin-D1, and Wnt3A in comparison to the CON group. However, SC06 administration was observed to reverse this trend (Figure 8B). Consistently, qPCR analysis further demonstrated that SC06 significantly (*p* < 0.05) upregulated the mRNA expression of Wnt/β-catenin signaling-related genes, including β-catenin, Cyclin-D1, AXIN, Wnt3, BMI-1, LRP5, LRP6, and FZD7, compared with the CP group (Figure 8C).

### 3.6. B. amyloliquefaciens SC06 Accelerates ISC Proliferation in Jejunum Organoids of Mouse

**Figure 9:**
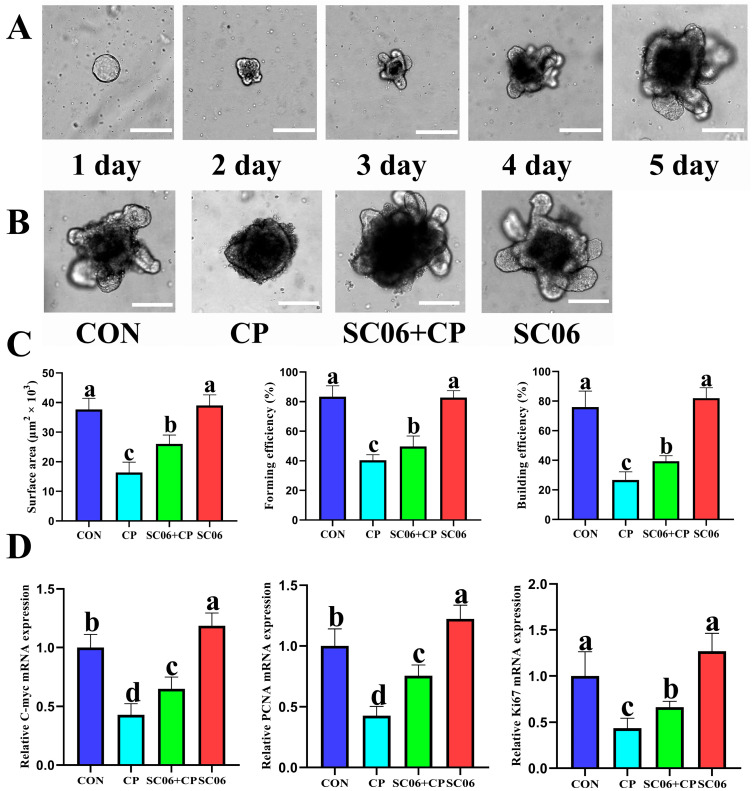
SC06 upregulated intestinal organoid proliferation under *C. perfringens*-challenge. (**A**) Crypts from small intestines were seeded onto Matrigel and cultured for 5 days to obtain well-developed organoids. (**B**) Organoids were treated with or without SC06 (1 × 10^8^ CFU per well) for 24 h, *C. perfringen* (1 × 10^8^ CFU per well) was utilized to create an inflammatory model for 6 h; Scale bars, 100 μm; (**C**) The surface area, forming efficiency and building efficiency of organoids was calculated. (**D**) he relative mRNA of *C-myc*, *PCNA* and *Ki67*. Means ± SD (n = 6). ^a–d^ Means within a row with various superscripts vary substantially (*p* < 0.05).

In previous experiments, we observed that SC06 facilitated the proliferation of ISCs following *C. perfringens*-induced injury. To ascertain whether SC06 expedites ISC proliferation, we conducted a co-culture experiment involving SC06 and intestinal organoids (Figure 9A). Following the continuous administration of *C. perfringens* (1 × 10^8^ CFU/mL) for a period of six hours, a significant reduction (*p* < 0.05) in both the area and budding rate of the organoids was observed in comparison to the control group. Nevertheless, pretreatment with SC06 led to a notable (*p* < 0.05) reversal of this trend, as evidenced by a statistically significant difference (Figure 9B,C). Furthermore, SC06 pretreatment was found to significantly (*p* < 0.05) enhance the expression of the *Ki67*, *PCNA* and *C-myc* genes in intestinal organoids subjected to a *C. perfringens* challenge (Figure 9D).

### 3.7. B. amyloliquefaciens SC06 Accelerated ISC Proliferation and Differentiation in C. perfringens-Challenged Organoid

**Figure 10:**
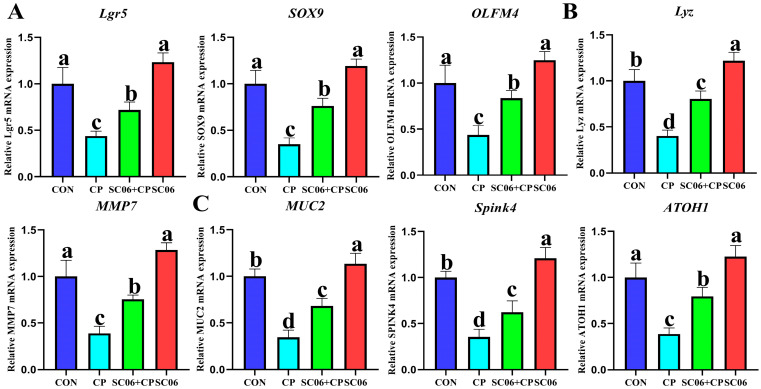
SC06 upregulated intestinal organoid differentiation under *C. perfringens*-challenge. (**A**) the relative mRNA of stem cell marker (*Lgr5*, *Sox-9* and *Olfm4*). (**B**) the relative mRNA of Paneth cell marker genes (*LYZ* and *MMP7*) (**C**) the relative mRNA of goblet cell marker (*MUC2*, *ATOH1*, and *Spink4*). Means ± SD (n = 6). ^a–d^ Means within a row with various superscripts vary substantially (*p* < 0.05).

The qPCR results demonstrated that, in comparison with the CON group, the *C. perfringens* significantly (*p* < 0.05) increased the mRNA expressions of active stem cell markers (*Lgr5*, *SOX9*, and *OLFM4*), goblet cell indicators (*MUC2*, *ATOH1*, and *SPINK4*), and paneth cell markers (*LYZ* and *MMP7*). Conversely, SC06 treatment reversed (*p* < 0.05) this trend (Figure 10).

### 3.8. Wnt Inhibition Attenuated the Protective Effect of B. amyloliquefaciens SC06 on C. perfringens–Challenged Jejunum Organoids

**Figure 11:**
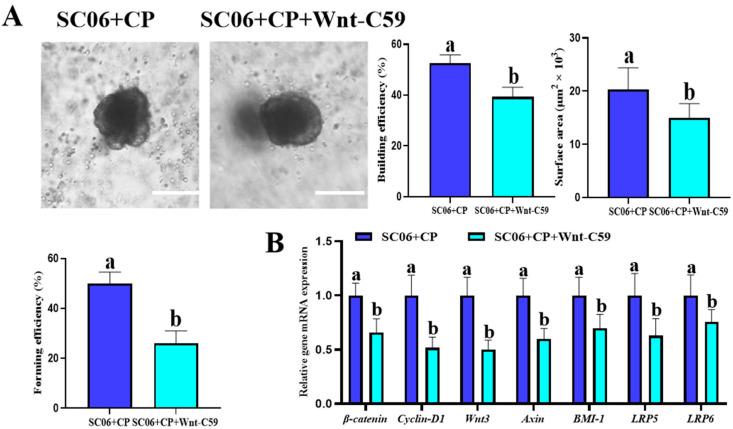
Effects of Wnt inhibition on the protective role of SC06 in intestinal organoids challenged with C. perfringens. SC06 + CP + Wnt-C59: Pre-treated with 100 nM Wnt-C59 for 24 h before SC06 and CP. (**A**) Representative images of intestinal organoids from the SC06 + CP group and the SC06 + CP + Wnt inhibitor group. Scale bars, 100 µm. Quantitative analysis showed that budding efficiency, surface area, and forming efficiency were significantly reduced following Wnt inhibition compared with SC06 + CP. (**B**) Quantification of organoid forming efficiency and relative mRNA expression of Wnt/β-catenin signaling–related genes (*β-catenin*, *Cyclin*-D1, *Wnt3*, *Axin*, *BMI-1*, *LRP5*, and *LRP6*). Data are presented as means ± SD (n = 6). ^a,b^ Means within a row with different superscripts differ significantly (*p* < 0.05).

As shown in Figure 11, treatment with a Wnt inhibitor significantly (*p* < 0.05) impaired the protective effects of SC06 on jejunum organoids. Compared with the SC06 + CP group, organoids in the SC06 + CP + Wnt inhibitor group exhibited decreased budding efficiency, reduced surface area, and lower forming efficiency. Moreover, the mRNA expression of Wnt/β-catenin signaling–related genes (*β-catenin*, *Cyclin-D1*, *Wnt3*, *Axin*, *BMI-1*, *LRP5*, and *LRP6*) was markedly (*p* < 0.05) downregulated following Wnt inhibition. These results indicate that the beneficial effects of SC06 on organoid growth and ISC-associated gene expression are largely dependent on the activation of the Wnt/β-catenin signaling pathway.

## 4. Discussion

Bacillus have been shown to be beneficial for intestinal health and have been employed to prevent and treat a variety of gastrointestinal disorders in numerous studies [24,25,26]. Our studies have shown that the administration of *B amyloliquefaciens* SC06 can effectively enhance the intestinal physical barrier in *C. perfringens*-challenged mouse by promoting the regeneration and proliferation of ISC through the activation of the Wnt/β-catenin signaling pathway. In the context of an organoid co-culture experiment, it was shown that the stimulatory impact of SC06 on the regeneration and proliferation of ISCs was facilitated via the Wnt/β-catenin signaling pathway. These findings suggest that SC06 may regulate the prefiltration and differentiation of ISCs into Paneth and goblet cells by activating the Wnt/β-catenin signaling pathway, thereby facilitating the repair of damaged intestinal epithelium.

In the present study, we selected *C. perfringens* type A (ATCC 13124) as the challenge strain because it is a well-characterized and widely available reference strain for modeling intestinal injury. Unlike many clinical isolates, *C. perfringens* type A does not carry additional virulence plasmids encoding spacial toxins such as NetB or β-toxin, which are strongly associated with poultry necrotic enteritis and human enteritis necroticans, respectively [27,28,29]. This feature ensures experimental reproducibility and facilitates mechanistic analysis of host–pathogen interactions without the confounding effects of multiple toxin profiles. Pathogenic differences between *C. perfringens* type A and virulent clinical isolates should, however, be noted. Whereas strains such as *C. perfringens* type C or *NetB-positive* type A strains induce intestinal necrosis primarily through toxin-mediated cytotoxicity, *C. perfringens* type A primarily elicits host immune-mediated responses, including inflammation, oxidative stress, and epithelial apoptosis. In this context, our findings that B. amyloliquefaciens SC06 supplementation attenuates inflammation, restores redox balance, and promotes intestinal stem cell regeneration are particularly relevant. Although our results do not directly establish SC06’s efficacy against toxin-mediated necrosis, they do demonstrate its capacity to mitigate the host immune response pathway damage that is common to *C. perfringens* infections. These findings highlight the potential of SC06 as a therapeutic adjunct for mucosal healing, and provide a valuable reference for future studies using virulent isolates in poultry or human disease models.

ATCC 13124 strain produces α-toxin (phospholipase C) and perfringolysin O, and the pathological features observed in our study can be understood in relation to their actions. α-toxin hydrolyzes membrane phospholipids and disrupts tight junction integrity, consistent with our findings of reduced Claudin, Occludin, and ZO-1 expression as well as increased serum D-lactic acid, DAO, and endotoxin [28,30,31]. Perfringolysin O, a cholesterol-dependent pore-forming cytolysin, activates inflammasome signaling and promotes IL-1β release, in line with the elevated pro-inflammatory cytokines (TNF-α, IL-1β, IL-6, IL-8) and M1 macrophage polarization we observed [28,31,32]. Both toxins can disturb mitochondrial homeostasis, thereby contributing to oxidative stress (increased MDA, decreased SOD, CAT, and GSH) and apoptosis of epithelial cells, as demonstrated by our TUNEL analysis [30,31]. Persistent inflammation and toxin-mediated epithelial injury further impair intestinal stem cell renewal, consistent with the downregulation of ISC markers and suppressed organoid proliferation in the CP group. These findings suggest that the epithelial damage, inflammatory response, oxidative stress, and impaired regeneration seen in our model are mechanistically linked to the actions of α-toxin and perfringolysin O.

The intestine plays a significant role in nutrient absorption [33,34], and the health of the villi is a crucial determinant of this process [35]. Prior research has indicated that *C. perfringens* has the potential to inflict damage upon the mucosa and the crypt-villus axis, resulting in the formation of numerous atrophic villi and a reduction in reproductive crypts [36,37]. Our studies demonstrated that SC06 exhibited increased villus height and V/H ratio in the small intestine. Wang et al. also found the *B. amyloliquefaciens* may improve the morphology of small intestine [38]. Impaired barrier function causes increased leaking of intraluminal antigens and toxins, which activates the gut immune response [39]. It has been demonstrated in multiple studies that immune stress or inflammation damage induced by *C. perfringens* can result in damage to the intestinal mucosa barrier in mice [40]. Our study demonstrated that SC06 supplementation suppressed the *C. perfringens*-induced elevations of TNF-α, IL-1β, IL-6, and IL-8. These cytokines are key mediators of mucosal injury: TNF-α and IL-1β promote epithelial apoptosis and barrier disruption, IL-6 sustains inflammatory signaling, and IL-8 drives neutrophil recruitment and oxidative damage. The reduction of these cytokines by SC06 therefore reflects not only an anti-inflammatory effect but also a shift toward an environment more permissive for epithelial repair and barrier restoration, which was consistent with previous results [41,42]. The serum levels of antioxidant enzymes reflected the host’s antioxidant capacity, which was collaboratively utilized to eradicate surplus free radicals and preserve homeostasis [43]. It was observed that *B. amyloliquefaciens* SC06 increased the activity of antioxidant enzymes (including GSH, CAT, SOD), while simultaneously decreasing the oxidative stress markers MDA content in *C. perfringens*-challenged mice. Inflammation and oxidative stress have been shown to trigger apoptosis both in vitro and in vivo [44]. The present investigation found that SC06 supplementation decreased intestinal apoptosis produced by *C. perfringens* exposure. Previous studies have also shown that oral treatment of *B. amyloliquefaciens* B10 dramatically reduces the apoptosis index in mice [45]. These findings suggested that an imbalance in apoptosis caused by *C. perfringens* might play a role in the development of intestinal inflammation and oxidative stress. In general, the results indicated that the supplementation of *B. amyloliquefaciens* SC06 may inhibit the apoptosis of epithelial cells by inhibiting enteric inflammation and oxidative stress.

The interaction between pathogenic bacteria and immune cells may result in damage to the intestinal epithelium, thereby allowing pathogenic bacteria to enter the mucosal layer and submucosa. This process may ultimately lead to the development of NE [46,47]. Consequently, the ability of the intestinal epithelium to repair itself is closely related to the process of mucosal healing in NE. Renewing and differentiating intestinal stem cells (ISCs) is necessary for the development of intestinal epithelial cells. It is very necessary for the healing of damaged intestinal epithelium to have a continuous regeneration of intestinal epithelial cells throughout the body [44,48]. In the context of tissue repair and homeostasis, ISCs serve as the primary driving force behind the maintenance of epithelial integrity [49]. Wang et al. [50] found that *Limosilactobacillus reuteri* DS0384 boosting intestinal stem cell activity and strengthening intestinal barrier integrity, which restored the injured intestinal epithelium, alleviating *C. sakazakii*-induced clinical signs and intestinal epithelial damage. Our results demonstrated that SC06 administration substantially elevated the mRNA levels of ISC maker genes in the *C. perfringens* challenged-mouse model. The results demonstrated that SC06 can preserve the structural integrity of the intestinal mucosa by facilitating the regeneration of intestinal stem cells (ISC). The mechanism by which goblet cells and Paneth cells protect against microbial invasion and harbor microbes within two layers of mucin is well known [51,52,53]. We found that SC06 treatment promoted the differentiation of intestinal stem cells into goblet cells and Paneth cells in both mouse models and organoids. The results of our study confirm that SC06 can regulate the differentiation and renewal of ISCs, thereby enhancing epithelial regeneration and repair in mice subjected to *C. perfringens* challenge.

In the presence of normal physiological circumstances, the process of ISC renewal and differentiation takes place within an in vivo environment that is referred to as the intestinal stem cell niche. This niche comprises signaling molecules and cell-to-cell contacts that regulate the activity of IS cells [54]. A comprehension of the mechanisms through which SC06 regulates niche signaling pathways in the context of inflammation could potentially transform the treatment of NE. Prior research has demonstrated that Wnt/β-catenin signaling is a vital mechanism for replenishing depleted ISCs and sustaining their normal functions [55,56]. Wnt/β-catenin signaling, which is critical for the fate of ISCs, is concentrated in the crypts and exhibits a progressive decrease along the crypt-villus axis [57]. The present study demonstrated that Wnt/β-catenin signal pathway-related genes exhibited a notable elevation in the jejunum of *C. perfringens*-challenged mice or organoids following SC06 supplementation. This finding offers insight into the potential molecular mechanism underlying the barrier-protective effects of SC06. Previous studies have shown evidence that some variables, such as deoxynivalenol, heat-stable enterotoxin, and heat exposure, have the ability to suppress the canonical Wnt/β-catenin pathway and the proliferation of intestinal stem cells (ISC) [58,59,60]. It was therefore postulated that SC06 modulates the differentiation and renewal of ISCs, thereby strengthening the intestinal physical barrier through the activation of the Wnt/β-catenin pathway. Intestinal organoids exhibit a capacity for replication that is analogous to that observed in crypt-villus structures, encompassing the ability to reproduce a diverse array of intestinal epithelial cell types [21]. Consequently, intestinal organoids offer a valuable research tool for investigating the mechanisms underlying ISC-mediated intestinal epithelial healing [61]. Ryu Nishimura et al. [62] developed human colon organoids with the objective of identifying a novel pharmaceutical target for the repair of damaged intestinal epithelium. Prior research has indicated that the incorporation of probiotics into ISCs facilitates intestinal epithelial regeneration [63]. A co-culture model of intestinal organoids was created to mimic the morphological properties of intestinal mucosa in vivo, with a special emphasis on the effect of SC06 on intestinal epithelial regeneration. The findings of this study indicate that SC06 has the capacity to alleviate the inflammatory damage caused by *C. perfringens* in jejunum organoids and to enhance the expression of ISC marker genes. Additionally, SC06 elevated the expression of goblet cell and paneth cell marker genes while reducing the number of enteroendocrine cells, enterocytes, and absorptive cells. This may facilitate the restoration of normal intestinal function. The results of the organoid experiment demonstrated that SC06 has the potential to facilitate the renewal and differentiation of ISCs, thereby promoting the healing of damaged intestinal epithelium. Prior research indicated that *L. reuteri* facilitated the growth of intestinal organoids by activating the Wnt/β-catenin signaling pathway [64]. The research found that SC06 activates the Wnt/β-catenin pathway, leading to increased gene and protein expression, which enhanced cell proliferation. SC06 improves *C. perfringens*-induced intestinal injury in mice by increasing ISC proliferation via the Wnt/β-catenin pathway.

This study has several limitations that should be noted. First, mice were not pretreated with antibiotics, and baseline microbiota composition was not analyzed. While this design provided a relatively stable background to evaluate the protective effects of SC06 against *C. perfringens*, it does not account for the influence of microbial competitors that may shape disease outcomes. Future studies incorporating microbiome sequencing and microbial manipulation models will be necessary to clarify how SC06 interacts with the gut microbiota to modulate infection and mucosal repair. Second, we evaluated intestinal barrier disruption using multiple complementary readouts—including serum D-lactic acid, diamine oxidase, and endotoxin levels, tight junction gene expression, and microvilli ultrastructure—in addition to histological analysis. These data provide functional support for the protective role of SC06 on barrier integrity. Nevertheless, direct permeability assays (e.g., FITC-dextran) or bacterial translocation experiments were not performed, and these approaches remain important future directions to validate and extend our observations.

## 5. Conclusions

In conclusion, SC06 demonstrated efficacy in mitigating intestinal mucosal damage and promoting ISC differentiation and regeneration in a *C. perfringens*-challenged mouse model, with the mechanism of action attributed to the Wnt/β-catenin pathway. In vitro testing demonstrated that SC06 enhanced *C. perfringens*-induced jejunum organoid proliferation and facilitated the healing of *C. perfringens*-injured organoids. Furthermore, the mechanism by which SC06 regulates ISCs has been shown to align with the results obtained in vivo. The data indicate that SC06 stimulates the renewal and differentiation of ISCs, thereby accelerating epithelial regeneration and repair, which suggests that SC06 may be a beneficial agent for mucosal healing in patients with neoplasia.

## Figures and Tables

**Table 1:** Sequences of the oligonucleotide primers used for quantitative real-time PCR.

Primer Name	Accession Numbers	Primer Sequence (5′-3′)
*Ki67*	NM_001081117.2	F: TCATTGACCGCTCCTTTAGGTATG
		R: TTGGTATCTTGACCTTCCCCA
*C-myc*	NM_010849.4	F: CGTTGGAAACCCCGCAGA
		R: TACGGAGTCGTAGTCGAGGT
*PCNA*	NM_011045.2	F: GGGTTGGTAGTTGTCGCTGT
		R: GATTCACGATGCAGAAGCGG
*Claudin-1*	NM_016674.4	F: CAACCCGAGCCTTGATGGTA
		R: TCATGCCAATGGTGGACACA
*Claudin-2*	XM_006528486.4	F: ACCTAGTCCTTGTCCCCAGAA
		R: GAAGGCATCTAGAAAACGACCAG
*Claudin-5*	NM_013805.4	F: CCCAGTTAAGGCACGGGTAG
		R: GGCACCGTCGGATCATAGAA
*β-catenin*	NM_001165902.2	F: CTCGTGTCCTGTGAAGCCC
		R: CAGGTCAGCTTGAGTAGCCA
*Wnt3A*	NM_009522.3	F: TCCTGTCTGGGATACGGGTT
		R: TGTCGGGTCAAGAGAGGAGT
*Cyclin-D1*	NM_001379248.1	F: CAACTTCCTCTCCTGCTACCG
		R: TGGAGGGGGTCCTTGTTTAG
*LRP5*	NM_008513.3	F: TGGACTGGATGGGCAAGAAC
		R: CCTGGGGTTGTCAAGGTCTC
*LRP6*	NM_008514.4	F: CTGCGGTGGACTTTGTGTTT
		R: CTCCAAGCCAATCACATGCC
*FZD7*	NM_008057.3	F: ACCCTACTGCTCCCTACCTG
		R: AGAAGGGGAAAGACAAGCGG
*BMI-1*	NM_001416911.1	F: TGCTGGAGAGCTGGAAAGTG
		R: GTGAGGGAACTGTGGGTGAG
*AXIN*	NM_001159598.2	F: CTTCAGGGCTCTGGGCTC
		R: AAAACCGGCTGCTCACTCTC
*Lgr5*	NM_010195.2	F: AATGCCTCTCCACACTTCGG
		R: ACATCGAACACCTGCGTGAA
*SOX9*	NM_011448.4	F: AGCACAAGAAAGACCACCCC
		R: CTCCGCTTGTCCGTTCTTCA
*OLFM4*	NM_001351947.1	F: GGCACGATGAGTTACAGCCT
		R: TTCTGCCCAAGAAGTGCCTC
*MUC2*	NM_023566.4	F: GAAGCCAGATCCCGAAACCA
		R: GAATCGGTAGACATCGCCGT
*ATOH1*	NM_007500.5	F: CCCGTCAAAGTACGGGAACA
		R: CTCGTCCACTACAACCCCAC
*Spink4*	NM_011463.2	F: CTTGGCATGGACAGGGAACT
		R: TGGTTTTCATCCGGGTCAGG
*LYZ*	NM_013590.4	F: GACTAGTGAGCTGTGCCTGT
		R: TGCTCCTGTGGTTATTGGCTG
*MMP7*	NM_001319986.2	F: TTGGGCAGAATGTTCCTGGTT
		R: TTTTCCAGTCATGGGCAGGC

## Data Availability

The original contributions presented in this study are included in the article/Supplementary Materials. Further inquiries can be directed to the corresponding authors.

## Supplementary Materials

The following supporting information can be downloaded at: https://www.mdpi.com/article/10.3390/microorganisms13092136/s1, Figure S1: Western blot raw data image.
