# Supplementary figures and images for: Bacillus amyloliquefaciens SC06 Ameliorated Intestinal Mucosal Injury by Regulated Intestinal Stem Cells Proliferation and Differentiation via Activating Wnt/β-Catenin Signal Pathway in Clostridium perfringens-Challenged Mouse
Source: Microorganisms. 2025 Sep 12;13(9):2136. doi: 10.3390/microorganisms13092136 (PMC12472280; doi:10.3390/microorganisms13092136)

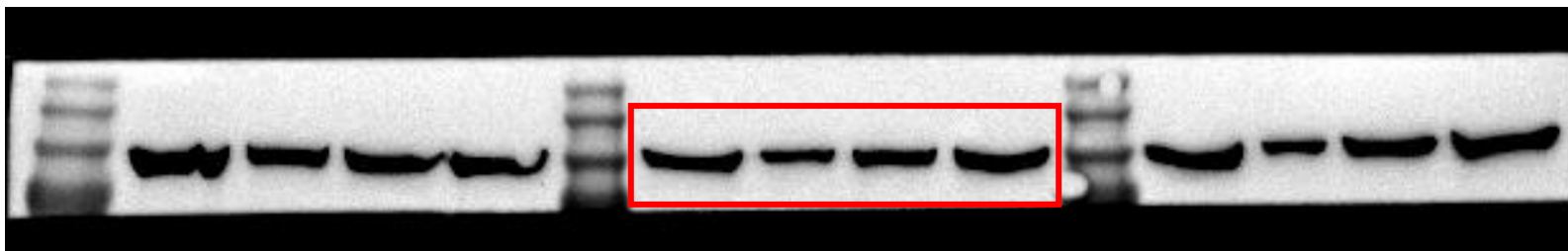

$\beta$ -catenin

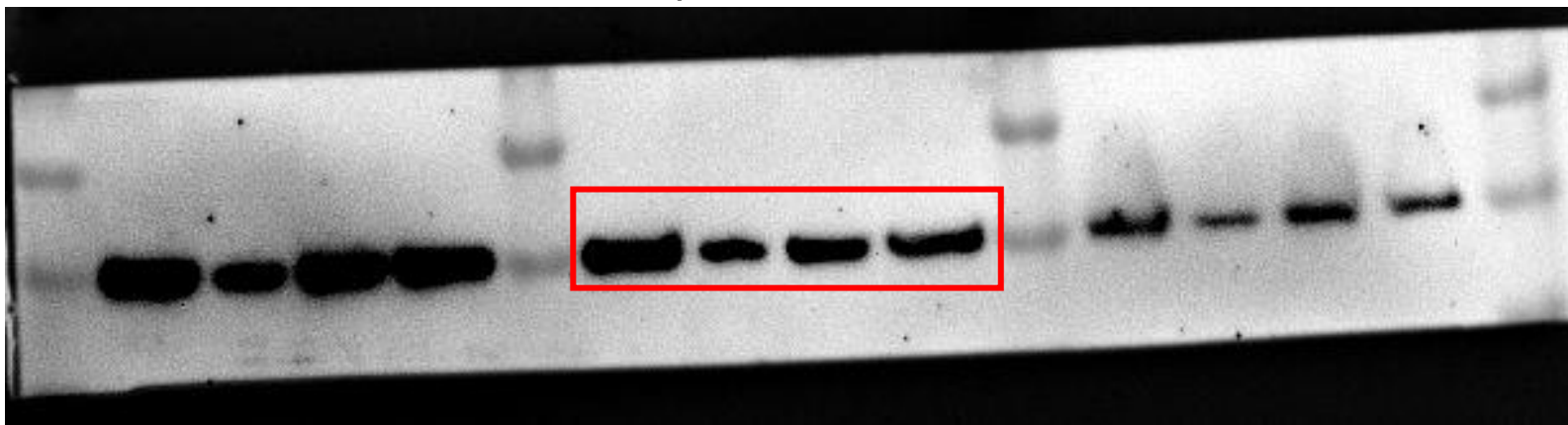

FZD7

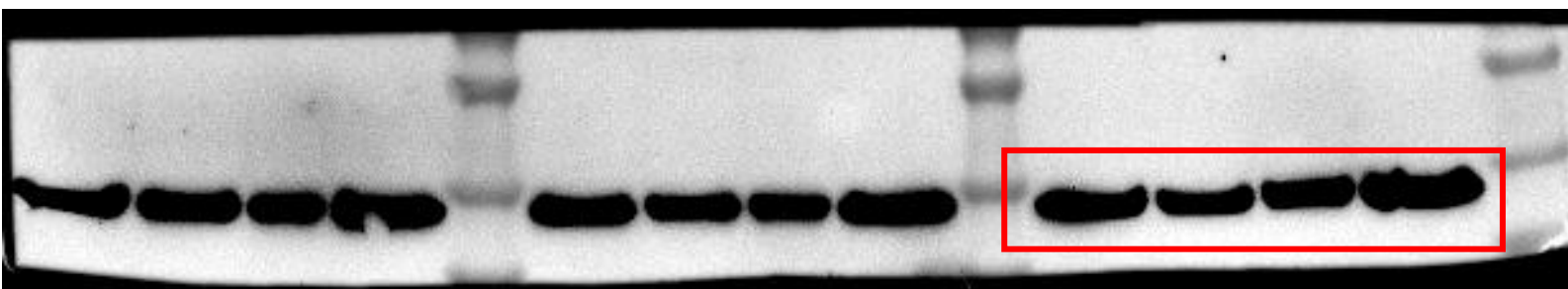

$\beta$ -actin

Supplement: Supplementary file 1 [file microorganisms-13-02136-s001.zip › microorganisms-3790133-supplementary.pdf]
